# Rewiring cell identity and metabolism to drive cardiomyocyte proliferation

**DOI:** 10.1186/s13619-025-00257-7

**Published:** 2025-09-28

**Authors:** Lixia Zheng, Yuanyuan Chen, Jing-Wei Xiong

**Affiliations:** 1 Beijing Key Laboratory of Cardiometabolic Molecular Medicine, and, Institute of Molecular Medicine, College of Future Technology, Peking University, Beijing, 100871 China; 2 School of Basic Medical Sciences, The Second Affiliated Hospital, Institute of Biomedical Innovation, and The MOE Basic Research and Innovation Center for the Targeted Therapeutics of Solid Tumors, Jiangxi Medical College, Nanchang University, Nanchang, 330031 China

**Keywords:** Heart regeneration, Cardiomyocyte proliferation, Dedifferentiation, Metabolic reprogramming, Swine

## Abstract

The adult mammalian heart exhibits minimal regenerative capacity due to postnatal cell-cycle arrest of cardiomyocytes. In contrast, lower vertebrates such as zebrafish retain the ability to fully regenerate heart after injury. This capacity is driven not only by transcriptional and structural plasticity but also by metabolic reprogramming that supports cardiomyocyte proliferation. Adult mammalian cardiomyocytes lack both features, remaining largely refractory to regenerative cues. These limitations have prompted efforts to identify extrinsic genetic and metabolic regulators capable of reactivating proliferative competence in adult cardiomyocytes. In this review, we highlight recent advances in the molecular and metabolic control of cardiomyocyte cell-cycle reentry, focusing on strategies that modulate dedifferentiation, proliferation, and redifferentiation as well as metabolic state transitions. We also examine emerging translational approaches in swine models, which more closely recapitulate human cardiac physiology than rodents. Together, these insights provide a roadmap for unlocking endogenous regenerative pathways and identify key challenges in translating these findings into therapies for heart failure.

## Background

Cardiovascular disease remains the leading cause of death worldwide, with myocardial infarction (MI) posing a major clinical challenge. Extensive cardiomyocyte (CM) loss following MI triggers fibrotic remodeling and progressive ventricular dysfunction, ultimately leading to heart failure (Murry et al. 2006). Although current therapies, including pharmacologic agents, percutaneous interventions, mechanical support, and transplantation can alleviate symptoms and delay disease progression, none can replenish lost CMs. In contrast to adult mammals, lower vertebrates such as zebrafish and salamanders retain a lifelong capacity for cardiac regeneration, primarily through injury-induced proliferation of pre-existing CMs (Poss et al. 2002; Senyo et al. 2013). In mammals, this regenerative potential is restricted to a brief postnatal window and is rapidly lost thereafter (Porrello et al. 2011). The low turnover rate and post-mitotic state of adult CMs remain key barriers to heart regeneration.

In regenerative zebrafish, mature CMs respond to injury by undergoing dedifferentiation and metabolic reprogramming (Honkoop et al. 2019). This transition enables CMs to acquire a more plastic, embryonic-like phenotype and shift their energy metabolism from oxidative phosphorylation to glycolysis, a metabolic state that supports cell-cycle reentry and tissue regeneration. Adult mammalian CMs, by contrast, lack this intrinsic plasticity and remain metabolically rigid after injury, failing to reactivate regenerative programs. Nonetheless, emerging evidence shows that various extrinsic factors, including transcription factors, microRNAs (miRNAs), small molecules and growth factors can regulate CM proliferation in adult mammals (Du et al. 2022; Zheng et al. 2021). Among these, several regulators can trigger dedifferentiation and metabolic rewiring, thereby mimicking key features of developmental and regenerative states (Abouleisa et al. 2022; Chen et al. 2021; Du et al. 2022). Notably, specific enzymes and metabolites within glycolysis and the tricarboxylic acid (TCA) cycle have been directly linked to CM proliferative capacity, reinforcing the critical interplay between metabolic state and regenerative competence (Cardoso et al. 2020; Cheng et al. 2022; Magadum et al. 2020). Together, these findings point to a tightly coupled network of dedifferentiation, metabolic reprogramming, and cell-cycle reentry as a core framework for cardiac regeneration and highlight the therapeutic potential of targeting these processes in the adult heart.

However, most of these discoveries have emerged from rodent models, which only partially recapitulate human cardiac physiology and disease complexity. To bridge this translational gap, large-animal models such as swine are increasingly used to evaluate regenerative strategies in systems that more closely mirror human biology. Several CM proliferation regulators have shown efficacy in swine, underscoring their translational relevance. Building on these advances, this review highlights recent progress in decoding the molecular and metabolic regulators of cardiomyocyte proliferation, examines their evaluation in large-animal models, and outlines outstanding challenges and future directions toward clinical translation.

## Molecular networks orchestrating dedifferentiation, proliferation and redifferentiation (DPR)

**Fig. 1:**
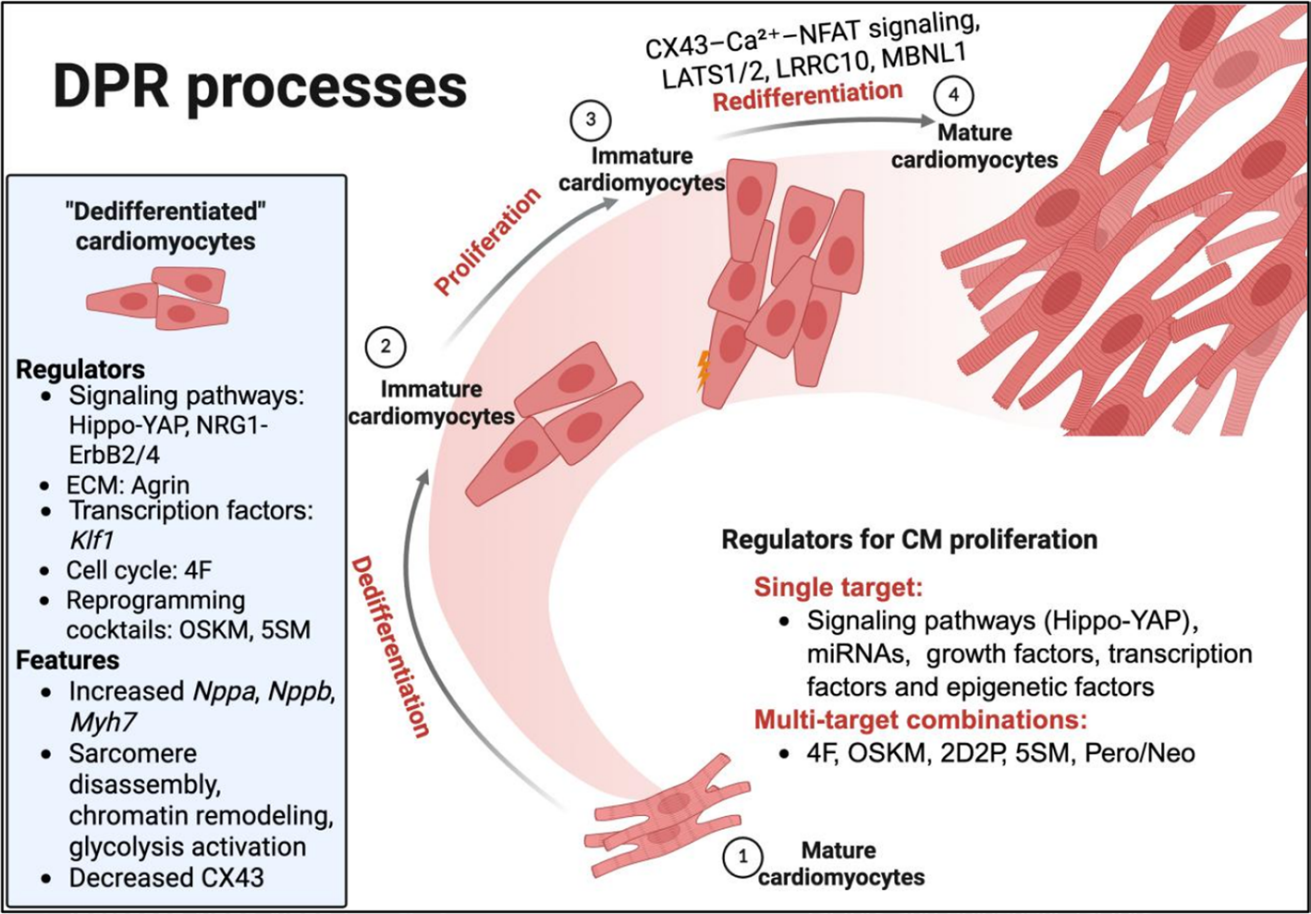
Schematic overview of cardiomyocyte regulators orchestrating the DPR process. Created with BioRender.com. Cardiac regeneration involves a sequential process of dedifferentiation, proliferation, and redifferentiation in pre-existing CMs. Mature CMs respond to injury by initiating dedifferentiation, marked by sarcomere disassembly, activation of cell-cycle genes, chromatin remodeling, and glycolysis reactivation. Key regulators include Hippo-YAP (Monroe et al. 2019) and NRG1-ErbB2/4 (Bersell et al. 2009) signaling, ECM proteins like Agrin (Bassat et al. 2017), transcription factors (*Klf1*) (Ogawa et al. 2021), cell-cycle drivers (4F) (Mohamed et al. 2018), and reprogramming cocktails (OSKM, 5SM) (Chen et al. 2021; Du et al. 2022). Dedifferentiated CMs exhibit increased expression of fetal and metabolic markers such as *Nppa*, *Nppb*, *Myh7*, and decreased CX43 expression (Zhu et al. 2021). Immature CMs then enter the proliferation phase, which is regulated by both single-target (e.g., signaling pathways, miRNAs, growth factors, epigenetic regulators) and multi-target strategies, including 4F (Mohamed et al. 2018), OSKM (Chen et al. 2021), 2D2P (Fu et al. 2024), 5SM (Du et al. 2022), and combined paromomycin/neomycin (Pero/Neo) (Ahmed et al. 2024). Redifferentiation restores contractile function and structural integrity in new CMs. This step is modulated by calcium signaling via CX43-mediated gap junctions, activation of the Ca^2^⁺–NFAT pathway (Wang et al. 2017), and feedback regulators such as LATS1/2 (Shakked et al. 2023), LRRC10 (Nguyen et al. 2023), and MBNL1 (Bailey et al. 2024). DPR: Dedifferentiation, proliferation, Redifferentiation; *Klf1*, Krüppel-like factor 1; OSKM, *Oct4*, *Sox2*, *Klf4*, *c-Myc*; 4F, CDK1, CCNB, CDK4, CCND; 5SM, phenylephrine hydrochloride, baricitinib, harmine, VO-OHpic trihydrate, AZD3965; CX43, connexin 43; LATS1/2, large tumor suppressor kinases 1/2; LRRC10, leucine-rich repeat-containing protein 10; MBNL1, muscleblind-like splicing regulator 1; 2D2P, *Tmsb4x*, *Tmsb10*, *Dmd*, and *Ctnna3*

Cardiac regeneration in adult zebrafish and neonatal mice is known to involve a coordinated sequence of DPR following myocardial injury (Jopling et al. 2010; Wang et al. 2017). In this process, terminally differentiated CMs transiently revert to an embryonic-like, plastic state, re-enter the cell cycle, and subsequently redifferentiate to restore myocardial structure and function. Increasing evidence suggests that each phase of DPR is orchestrated by a multifaceted regulatory network (Fig. 1).

### Mechanistic insights into cardiomyocyte dedifferentiation

Dedifferentiation, in which mature CMs partially revert to an embryonic-like, plastic state is a critical initiating event. However, the molecular and cellular characteristics that define this state remain incompletely understood. Multiple regulators have been implicated in CM dedifferentiation, including signaling pathways (Monroe et al. 2019), transcription factors (Ogawa et al. 2021), extracellular matrix components (Bassat et al. 2017), cell cycle regulators (Mohamed et al. 2018) and reprogramming cocktails (Chen et al. 2021; Du et al. 2022). These regulators collectively drive hallmark features of dedifferentiation: upregulation of cell-cycle genes, sarcomere disassembly, re-expression of embryonic markers (*Nppa*, *Nppb*, *Myh7*), metabolic remodeling, and suppression of structural proteins like Connexin 43 (CX43) (Zhu et al. 2021). Mechanistically, many of these regulators converge on a few conserved downstream pathways. For instance, YAP, ERK, and AKT have been shown to mediate dedifferentiation in response to various upstream signals (D'Uva et al. 2015; Ikeda et al. 2019). These cascades act through diverse mechanisms, including modulation of chromatin accessibility (Monroe et al. 2019; Ogawa et al. 2021), actin polymerization (Morikawa et al. 2017), and reactivation of fetal transcriptional programs (Monroe et al. 2019). Within the Hippo pathway, Mammalian sterile 20-like kinase (MST), Large tumor suppressor kinase (LATS), and Salvador (SALV) serve as upstream kinases that inhibit dedifferentiation by repressing YAP, Transcriptional enhanced associate domain 1 (TEAD1), and Oncostatin M (OSM) (Ikeda et al. 2019). Once released from this inhibition, YAP orchestrates a broad pro-dedifferentiation program encompassing cytoskeletal remodeling, chromatin loosening, and mitotic gene activation (Monroe et al. 2019; Morikawa et al. 2017). This transcriptional plasticity is complemented by structural and signaling cues. In zebrafish, for example, Krüppel-like factor 1 (*Klf1*) facilitates dedifferentiation and proliferation by repressing core cardiac transcription factors such as *Mef2, Gata4, and Nkx2.5* (Ogawa et al. 2021). Similarly, the ECM protein Agrin and NRG1-ErbB2/4 signaling enhance YAP, ERK, and AKT activation to initiate dedifferentiation (Bassat et al. 2017; D'Uva et al. 2015). In addition to single-pathway modulation, multi-target reprogramming cocktails have been developed to enhance dedifferentiation. These include CDK1, CCNB1, CDK4, CCND1 (4F) (Abouleisa et al. 2022), *Oct4*, *Sox2*, *Klf4*, and *c-Myc* (OSKM) (Chen et al. 2021), and Phenylephrine hydrochloride, Baricitinib, Harmine, VO-OHpic trihydrate, and AZD3965 (5SM) (Du et al. 2022), all of which promote dedifferentiation phenotypes such as sarcomere disassembly and increased dedifferentiation markers. Interestingly, our group found that 5SM activates dedifferentiation independently of both Hippo-YAP and NRG1-ErbB2/4 pathways, suggesting the existence of previously unrecognized mechanisms.

### Single- versus multi-target approaches for regulating CM proliferation

Over the past two decades, substantial progress has been made in elucidating the mechanisms that drive injury induced CM proliferation. Among the most well-studied pathways is the Hippo-Yes-associated protein (YAP) signaling cascade, initially recognized for its roles in embryonic development and organ size control. Core components of this pathway, including MST, LATS, and SALV have emerged as critical regulators of cardiac regeneration (Heallen et al. 2013). Beyond its direct mitogenic effects, Hippo-YAP signaling integrates signals from a variety of upstream pathways and cross-talks with other cardiac networks to modulate regenerative outcomes (Bassat et al. 2017; Heallen et al. 2011; Lin et al. 2015). To dissect the regenerative potential of YAP activation, the Martin laboratory employed multi-omics and biochemical approaches to probe its downstream mechanisms. Their work demonstrated that overexpression of a constitutively active YAP mutant, YAP5SA, reprograms adult CMs into an embryonic-like, proliferative state (Monroe et al. 2019). Within this context, they identified a YAP-responsive subpopulation termed aCM2, defined by heightened YAP activity and regenerative potential. These aCM2 cells engage in niche-like interactions with complement-expressing fibroblasts and C3AR1^+^ macrophages, resembling the pro-regenerative microenvironment seen in neonatal hearts (Li et al. 2024). This work highlights the critical role of YAP5SA in promoting cardiac regeneration via specialized cellular niches composed of regenerative CM progenitors and supportive non-CM populations. More recently, the same group uncovered a post-translational mechanism by which YAP activity is modulated through lysine acetylation. Specifically, acetylation at lysine 265 (K265) enhances YAP’s association with cytoskeletal components such as TUBA4A, resulting in its cytoplasmic retention and inhibition of cardiac regeneration following MI. Conversely, deacetylation at this site facilitates nuclear translocation of YAP, markedly boosting regenerative capacity (Liu et al. 2025). These findings underscore the importance of post-translational modifications in fine-tuning YAP-mediated regeneration and suggest that modulating its subcellular localization may represent a viable therapeutic strategy.

Beyond the Hippo-YAP axis, a diverse of molecular regulators has been implicated in driving CM proliferation. These include additional signaling pathways (Bersell et al. 2009; Campa et al. 2008; Liu and Zhong 2017), cell cycle modulators (Cheng et al. 2007; Mohamed et al. 2018), miRNAs (Gabisonia et al. 2019; Gao et al. 2019; Hullinger et al. 2012), growth factors (Engel et al. 2006), transcription factors (Chen et al. 2021; Malek et al. 2017), and epigenetic regulators (Xiao et al. 2016). Collectively, these findings support the concept that CM proliferation is orchestrated by a complex network of converging signals and effectors, rather than by single-gene programs. This idea is reinforced by growing evidence that multi-target strategies show a robust effect on stimulating cardiomyocyte proliferation. For example, the 4 F significantly boosts CM proliferation and improves cardiac function in both murine and porcine models of MI and ischemia–reperfusion (IR) injury (Abouleisa et al. 2022; Mohamed et al. 2018). Similarly, forced expression of OSKM promotes CM dedifferentiation and proliferation, although sustained activation raises concerns about tumorigenesis (Chen et al. 2021). A separate strategy involving actin cytoskeleton remodeling using a combination of *Tmsb4x*, *Tmsb10*, *Dmd*, and *Ctnna3* (2D2P) has also been shown to stimulate CM proliferation and support myocardial repair (Fu et al. 2024).

Despite their promise, gene-based multi-target strategies raise important concerns, including risks of uncontrolled gene expression, immune activation, and tumorigenicity. In contrast, small-molecule approaches offer several advantages, such as lower immunogenicity, ease of delivery, dose controllability, and reduced manufacturing cost. Our group recently identified a five-compound small-molecule cocktail, 5SM that robustly induces cell-cycle re-entry and cytokinesis in adult CMs, leading to improved cardiac regeneration and function in mouse and rat models of MI and IR injury (Du et al. 2022; Zheng et al. 2023). In addition, two FDA-approved aminoglycoside antibiotics, paromomycin (Paro) and neomycin (Neo), were shown to suppress *Meis1* and *Hoxb13*, thereby promoting CM mitosis and restoring cardiac function post-injury (Ahmed et al. 2024). Together, these findings underscore the therapeutic potential of small-molecule-based multi-target strategies for cardiac repair. However, their broader application faces challenges such as complex pharmacokinetics, potential off-target effects, and the need for precise timing and combination of agents. Addressing these limitations will be essential for translating these approaches into clinically viable therapies.

### Molecular and structural regulators of cardiomyocyte redifferentiation

Redifferentiation process is essential for restoring mature CM identity and contractile function following injury. Prolonged dedifferentiation and proliferation can result in pathological outcomes such as impaired contractility and cardiac hypertrophy, underscoring the need for timely and efficient redifferentiation. Emerging studies have begun to address this gap. For instance, co-culture of adult mouse CMs with neonatal rat CMs demonstrated that neonatal cells transmit calcium (Ca^2^⁺) signals through CX43 gap junctions, activating the calcineurin-NFAT pathway and enhancing contractility in adult CMs, highlighting the importance of intercellular communication during redifferentiation (Wang et al. 2017). Intrinsic feedback mechanisms also contribute to redifferentiation. LATS1/2 kinases, core components of the Hippo pathway activated during dedifferentiation, help reestablish CM maturity, offering insight into how dynamic signaling transitions govern regenerative timing (Shakked et al. 2023). Structural remodeling likewise plays a pivotal role. The cardiac dyad, a specialized site for calcium handling and excitation–contraction coupling, must be reassembled during redifferentiation. Leucine-rich repeat-containing 10 (LRRC10), a negative regulator of proliferation, facilitates dyad reformation following injury. Its downregulation promotes dedifferentiation and proliferation, while re-expression is necessary for functional maturation (Nguyen et al. 2023). At the transcriptional level, recent work has implicated muscleblind-like 1 (MBNL1), a protein co-expressed with mature cardiac gene programs, as a key mediator of redifferentiation. Its activity is regulated through the *Meis1*, calmodulin phosphatase axis, linking transcriptional control to calcium-dependent signaling cascades (Bailey et al. 2024).

## Metabolic regulation of cardiomyocyte proliferation and regeneration

### Cross-species comparison of cardiac metabolic tansitions

To better understand how metabolic state influences cardiac regenerative capacity, it is informative to examine the natural diversity of metabolic programming across species and developmental stages. Comparative studies have revealed distinct patterns of substrate utilization that closely correlate with regenerative potential in both lower vertebrates and mammals (Table 1). Zebrafish possess a remarkable capacity for cardiac regeneration, largely supported by a glycolytic metabolic program adapted to their chronically hypoxic aquatic environment. Proteomic analyses have shown that adult zebrafish ventricles express higher levels of fatty acid oxidation (FAO) enzymes compared to juveniles (Fukuda et al. 2020), indicating a modest postnatal metabolic transition. Yet, following cardiac injury, border zone cardiomyocytes re-induce glycolytic genes, suggesting a preferential reliance on glycolysis to support proliferation and maintain regenerative competence (Honkoop et al. 2019). This reactivation of an embryonic-like metabolic state aligns with their sustained regenerative ability.

**Table 1:** Comparison of cardiac metabolism across species and developmental stages

Species	Developmental Stage	Metabolic Substrates	Metabolic Characteristics	References
Zebrafish	Embryonic	Glucose	Glycolysis	Fukuda et al. 2020; Honkoop et al. 2019
	Adult	Fatty acids	Fatty acid oxidation	
Mouse	Embryonic	Glucose	Glycolysis	Karwi et al. 2018
	Early Postnatal (P1)	Fructose and glucose	Glycolysis	
	Late Postnatal (P7)	Fatty acids	Fatty acid oxidation	
Pig	Early Postnatal (< 1 d)	Glucose and lactate	Have fatty acid oxidation capacity	Ascuitto et al. 1989
	Early Postnatal (3d)	Fatty acids and lactate	Enhanced fatty acid oxidation	
	Late Postnatal (6–12d)	Primarily fatty acids	Fatty acid oxidation becomes main energy source	
Sheep	Fetal Stage	Glucose and lactate	Glycolysis	Bartelds et al. 1998, 1999; Fisher et al. 1980
	Early Postnatal (1–4d)	Increased fatty acid use, lactate still dominant	Primarily glycolysis; limited fatty acid oxidation	
	Late Postnatal (7w)	Fatty acids	Fatty acid oxidation becomes predominant	
Rabbit	Early Postnatal (P1)	Glucose and lactate	Primarily glycolysis; limited fatty acid oxidation	Itoi and Lopaschuk 1993; Lopaschuk et al. 1991
	Mid Postnatal (P7)	Fatty acids and lactate	Increased fatty acid oxidation; reduced glycolysis	
	Late Postnatal (P14)	Fatty acids	Fatty acid oxidation becomes predominant	

Strikingly, mammalian fetal hearts exhibit a comparable glycolytic phenotype, driven by in utero hypoxia and a placenta-derived substrate supply rich in glucose and lactate (Fisher et al. 1980; Lopaschuk et al. 1992). However, within days after birth, mammalian hearts undergo a rapid metabolic switch, marked by suppression of glycolysis and a robust induction of mitochondrial FAO ultimately supplying approximately 95% of ATP in the adult heart (Doenst et al. 2013; Karwi et al. 2018). This transition is accompanied by mitochondrial maturation, increased reactive oxygen species (ROS), and activation of the DNA damage response, culminating in cardiomyocyte cell-cycle exit and loss of regenerative potential (Lopaschuk and Jaswal 2010). Supporting this, multi-omics profiling of mouse hearts from postnatal day 1 (P1) to P7 revealed a shift from fructose-fueled glycolysis to oxidative phosphorylation, along with increased expression of β-oxidation enzymes, acylcarnitine transporters, and markers of oxidative stress (Karwi et al. 2018).

Comparable transitions occur in larger mammals. In lambs, fetal hearts derive approximately 48% of energy from glucose and close to 36% from lactate, with negligible FAO. By P4, fatty acid availability increases tenfold, initiating FAO as glucose oxidation declines. By 7 weeks, FAO becomes the dominant energy source, reflecting maturation of the oxidative phenotype (Bartelds et al. 1998; Bartelds et al. 1999). Similarly, rabbit hearts rely on glycolysis and lactate at P1, but switch to FAO by P14, coinciding with the cessation of proliferative potential (Itoi and Lopaschuk 1993; Lopaschuk et al. 1991). In neonatal pigs, FAO initiates within 24 h and increases progressively through the second postnatal week, though full metabolic maturation lags behind adult levels (Ascuitto et al. 1989). Notably, pigs exhibit an earlier and faster FAO transition than mice, underscoring interspecies variability in the timing of metabolic programming. Collectively, these cross-species comparisons highlight the central role of metabolic plasticity in regulating cardiomyocyte proliferation and suggest that targeting substrate utilization may offer a promising route to reactivate regeneration in the adult mammalian heart.

### Metabolic modulators that promote cardiomyocyte proliferation

**Fig. 2:**
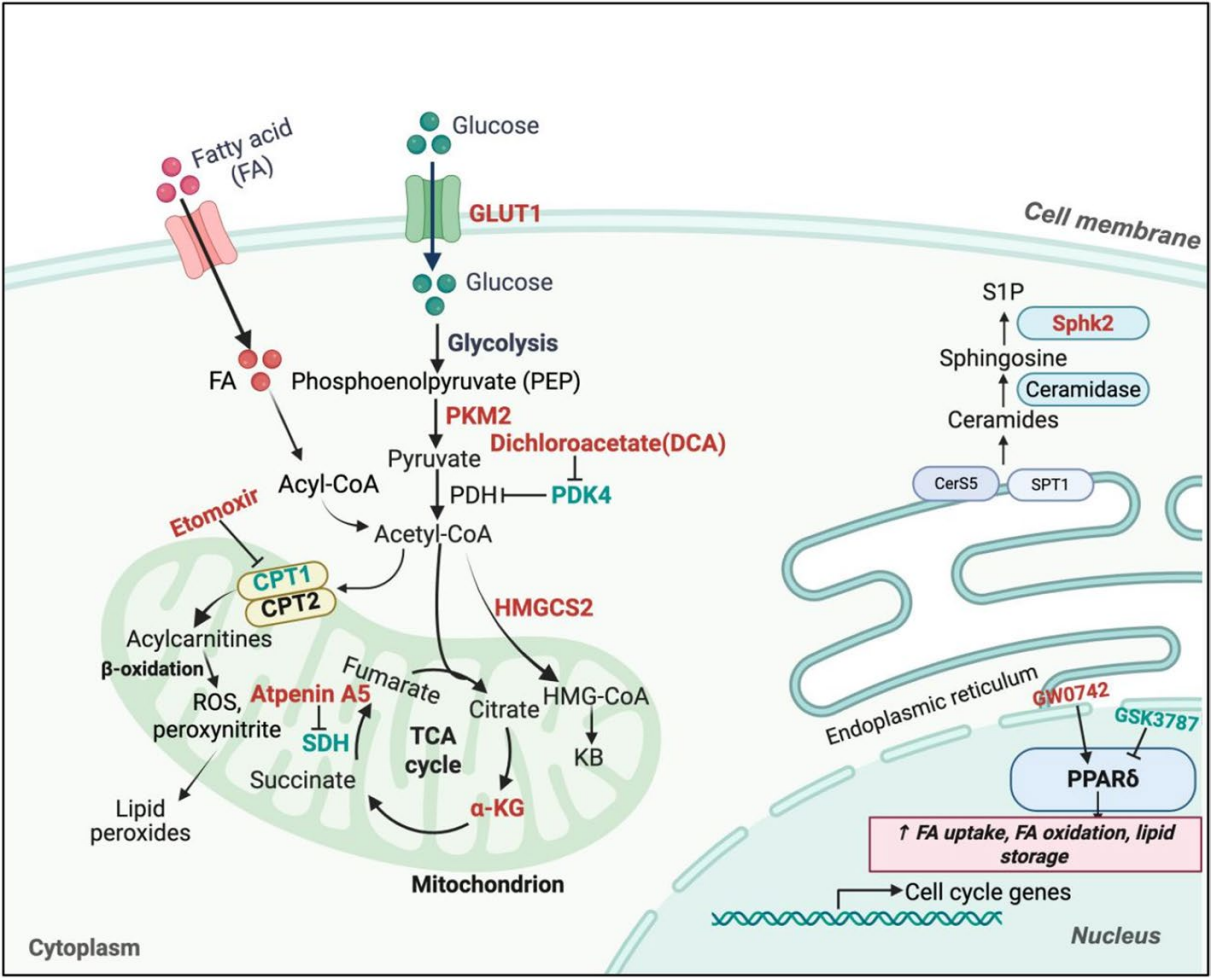
Metabolic regulation of cardiomyocyte proliferation. Metabolic reprogramming orchestrates CM proliferation during cardiac regeneration through multiple interconnected pathways, including glycolysis (e.g., PKM2, GLUT1, PDK4) (Magadum et al. 2020; Magadum et al. 2020; Cardoso et al. 2020), fatty acid oxidation (e.g., CPT1, PPARδ) (Li et al. 2023; Tang et al. 2025; Magadum et al. 2017), ketogenesis (e.g., HMGCS2) (Cheng et al. 2022)., sphingolipid metabolism (e.g., Sphk2) (Li et al. 2023), and the TCA cycle (e.g., α-KG, SDH) (Shi et al. 2024; Bae et al. 2021). Red-labeled regulators promote CM proliferation, while green-labeled factors inhibit it. FA, fatty acid; GLUT1, glucose transporter 1; PKM2, pyruvate kinase muscle isozyme 2; DCA, dichloroacetate; PDK4, pyruvate dehydrogenase kinase 4; CPT1, carnitine palmitoyltransferase 1; HMGCS2, 3-hydroxy-3-methylglutaryl-CoA synthase 2; SDH, succinate dehydrogenase; α-KG, α-ketoglutarate; Sphk2, sphingosine kinase 2; PPARδ, peroxisome proliferator-activated receptor delta

Emerging evidence highlights metabolic reprogramming as a central driver of CM proliferation during regeneration, functioning not merely as an energy source but as a dynamic regulator of chromatin architecture and transcriptional networks. Key metabolic pathways, including glycolysis (Fukuda et al. 2020; Magadum et al. 2020), FAO (Cardoso et al. 2020; Li et al. 2023), ketogenesis (Cheng et al. 2022), the TCA cycle (Shi et al. 2024) and sphingolipid metabolism (Ji et al. 2024) interface with epigenetic and transcriptional machinery to govern CM cell cycle re-entry (Fig. 2) (Table 2).

**Table 2:** Metabolic-epigenetic regulation of cardiomyocyte proliferation

Metabolic pathway	Key regulators	Manipulation strategy	Model	Epigenetic mechanism	Effect on cardiomyocyte state	Reference
Glucose metabolsim	Pkm2	Overexpression	P1 and adult mice	Non-enzymatic: Binds β-catenin to activate Cyclin D1, *c-Myc* Enzymatic: Upregulates G6PD to enhance PPP	Promotes proliferation and reduces oxidative stress	Magadum et al. 2020
	Pkm2a	Inhibition	adult zebrafish	/	Inhibits proliferation and reduces dedifferentiation	Huang et al. 2023
	Glut1	Overexpression	P7 mice	Increased nucleotide biosynthesis	Promotes cardiomyocyte proliferation	Fajardo et al. 2021
	PDK4	Inhibition	Adult mice	/	Shifts metabolism toward glucose oxidation, reduces ROS and DNA damage, reactivates cell cycle programs	Cardoso et al. 2020
	pdk3b	Overexpression	Adult zebrafish	/	Promotes proliferation and dedifferentiation	Fukuda et al. 2020
Fatty acid oxidation	CPT1B	Inhibition	Adult mice	α-KG activates KDM5, reducing H3K4me3 marks on maturation genes	Promotes proliferation and dedifferentiation, reduces DNA damage	Li et al. 2023
	CPT1	Inhibition	P7 and adult mice	CPT1 inhibition suppresses p38 MAPK phosphorylation mediated by ADP-ribosylation of DUSP1	Promotes cardiomyocyte proliferation	Tang et al. 2025
		Inhibition	P2 mice	/	Promotes cardiomyocyte proliferation and inhibit maturation	Cao et al. 2019
	Ppargc1a	Inhibition	Adult zebrafish	/	Promotes dedifferentiation and proliferation	Fukuda et al. 2020
	PPARδ	Overexpression	Adult zebrafish and adult mice	/	Promotes proliferation	Magadum et al. 2017
Ketogenesis	HMGCS2	Overexpression	Adult mice	/	Promotes dedifferentiation and proliferation	Cheng et al. 2022
Sphingolipid metabolism	Sphk2	Overexpression	P3 and adult mice	Histone acetylation via nuclear S1P on Erbb4, Mef2a, Mef2c promoters	Promotes proliferation	Ji et al. 2024
TCA Cycle	α-KG	Overexpression	P7 and adult mice	JMJD3-mediated H3K27me3 demethylation on cell cycle gene promoters	Promotes proliferation	Shi et al. 2024
	SDH	Inhibition	P1 and adult mice	/	Promotes CM proliferation, glycolysis and reduced DNA damage	Bae et al. 2021

Beyond its canonical role in energy production, metabolism actively orchestrates the molecular programs that define cardiomyocyte regeneration. Within the glycolytic axis, pyruvate kinase M2 (PKM2) exerts dual roles: catalyzing pyruvate production and translocating to the nucleus, where it partners with β-catenin to activate proliferative gene programs. In parallel, PKM2 diverts intermediates into the pentose phosphate pathway (PPP), fueling nucleotide synthesis and redox homeostasis, which is critical for mitotic progression and genome stability (Magadum et al. 2020). Overexpression of glucose transporter 1 (GLUT1) similarly enhances PPP flux and expands the Tnnt2^low^ CM population with high proliferative potential (Fajardo et al. 2021).

In zebrafish, cardiac injury induces expression of *Pkm* and *Pdk* genes in border-zone CMs, underscoring the conserved pro-regenerative role of glycolysis. Notably, overexpression of pyruvate dehydrogenase kinase (PDK) 3 enhances glycolysis and proliferation, while its inhibition impairs regeneration (Fukuda et al. 2020).

Conversely, postnatal induction of FAO poses a metabolic barrier to proliferation. Inhibition of Carnitine palmitoyltransferase 1B (CPT1B), the rate-limiting enzyme in mitochondrial fatty acid uptake, elevates intracellular α-ketoglutarate (α-KG), which activates Lysine (K)-specific demethylase 5 (KDM5)-mediated H3K4me3 demethylation at maturation loci, promoting a dedifferentiated state (Li et al. 2023). FAO blockade also downregulates Poly (ADP-ribose) polymerase 1 (PARP1), reducing p38 MAPK activation and releasing a key checkpoint on CM cycling (Tang et al. 2025). Deletion of PDK4 reprograms metabolism toward glucose oxidation, attenuates DNA damage signaling, and restores proliferation in neonatal and adult CMs post-infarction (Cardoso et al. 2020).

Peroxisome proliferator-activated receptor (PPAR) signaling further illustrates the plasticity of metabolic control. PPARα activation promotes terminal maturation via FAO, whereas PPARδ activation, either genetically or pharmacologically, induces CM proliferation and limits post-injury fibrosis (Magadum et al. 2017; Fukuda et al. 2020).

Beyond conventional fuels, emerging metabolic regulators such as ketone bodies and sphingolipids modulate CM fate. β-Hydroxybutyrate, generated via 3-hydroxy-3-methylglutaryl-CoA synthase 2 (HMGCS2), promotes dedifferentiation and proliferation, though its epigenetic mechanisms remain incompletely defined (Cheng et al. 2022). Nuclear sphingosine-1-phosphate (S1P), synthesized by sphingosine kinase 2 (Sphk2), enhances histone acetylation at regenerative loci including *Erbb4*, *Mef2a*, and *Mef2c*, reinforcing a pro-proliferative transcriptional program (Li et al. 2023).

Within the TCA cycle, α-KG serves not only as a bioenergetic intermediate but also as a cofactor for Jumonji domain-containing protein 3 (JMJD3), facilitating H3K27me3 demethylation and reactivation of cell cycle genes (Shi et al. 2024). In contrast, succinate dehydrogenase (SDH) acts as a metabolic brake. SDH inhibition via malonate suppresses ROS production and relieves oxidative constraints on proliferation (Bae et al. 2021).

In summary, intermediates from glycolysis, FAO, ketogenesis, sphingolipid metabolism and the TCA cycle converge on the chromatin landscape to regulate regenerative competence. Targeted control of metabolic flux thus represents a promising therapeutic avenue. Future work should focus on mapping the spatiotemporal dynamics of metabolic signals and integrating these with transcriptional and epigenetic landscapes to enable precise modulation of heart regeneration.

### Delivery strategies for metabolic reprogramming in cardiomyocyte proliferation

Metabolic reprogramming has emerged as a promising strategy to stimulate CM proliferation and promote cardiac regeneration. Central to this approach is the precise, context-dependent delivery of metabolic modulators that ensures temporal control and cell-type specificity. Recent advances have introduced a wide array of delivery platforms (Table 3).

**Table 3:** Delivery strategies of metabolic modulators to regulate cardiomyocyte proliferation

Metabolic target	Delivery strategy	Effect on CM proliferation	Delivery route	Dosage & treatment window	Model	Reference
Increase PKM2	modRNA	Promote	Intramyocardial	3 injections, 20 μL each	Adult mice	Magadum et al. 2020
Increase G6PD	modRNA	Promote	Intramyocardial	3 injections, 20 μL each	Adult mice	
Activate SDH	dimethyl succinate	Inhibit	Intravenous and intraperitoneal	100 mg/kg for 7 days	P1 neonatal mice	Bae et al. 2021
Inhibit SDH	dimethyl malonate	Promote	Intraperitoneal	100 mg/kg for 14 days or 4 weeks	P1 neonatal and adult mice	
Inhibit SDH	Atpenin A5	Promote	Intraperitoneal	100 μg/kg for 14 days	P1 neonatal mice	
Inhibit Cpt1	Etomoxir	Promote	Intraperitoneal	20 mg/kg/day for 1 week in P7 and 2 weeks in adult mice	P7 neonatal and adult mice	Tang et al. 2025
	Etomoxir	Promote	Intraperitoneal	15 µg/g/day for 3 days in P2 mice	P2 neonatal mice	Cao et al. 2019
Activate PPARδ	GW0742	Promote	subcutaneous injection	1 mg/kg in adult mice for 14 days	Adult mice	Magadum et al. 2017
Inhibit PPARδ	GSK3787	Inhibit	Kept in aquarium water	5 μM for a duration of 24 h	Adult zebrafish	
Increase HMGCS2	AAV9-mediated HMGCS2	Promote	Intramyocardial and tail injection	/	Adult mice	Cheng et al. 2022
Increase Sphk2	AAV9-mediated Sphk2	Promote	Intramyocardial	a dose of 1 × 10^12^ viral particles per animal (approximately 50 μL)	P3 neonatal mice and adult mice	Ji et al. 2024
Increase α-KG	α-KG supplementation	Promote	Intraperitoneal	300 mg/kg/day for 2 weeks	P7 neonatal and adult mice	Shi et al. 2024
Inhibit PDK4	DCA	Promote	Gavage	0.44 mg/g every 12 h for 14 days	Adult mice	Cardoso et al. 2020
Inhibit PDK	DCA	Inhibit	Intraperitoneal	1 nmol/mg from 1 to 4 dpci	Adult zebrafish	Fukuda et al. 2020
Inhibit Glycolysis	2-DG	Inhibit	Intraperitoneal	1 mg/g from days 3 to 6, one more injection on day seven after injury	Adult zebrafish	Honkoop et al. 2019
	2-DG	Inhibit	Intraperitoneal	0.5 nmol/mg from 1 to 4 dpci	Adult zebrafish	Fukuda et al. 2020

Small molecules offer reversible, dose-titratable modulation of metabolic pathways. For instance, dichloroacetate (DCA), a PDK inhibitor, enhances PDH activity and glucose oxidation when administered via gavage in adult mice, thereby promoting CM proliferation after myocardial injury. However, intraperitoneal delivery of DCA in adult zebrafish fails to elicit similar effects, highlighting species-specific pharmacodynamics (Cardoso et al. 2020; Fukuda et al. 2020). Etomoxir, a CPT1B inhibitor, administered intraperitoneally in both neonatal and adult mice, suppresses FAO, leading to α-KG accumulation and histone demethylation, two synergistic events that drive CM dedifferentiation and cell cycle re-entry (Cao et al. 2019; Tang et al. 2025). Similarly, subcutaneous injection of GW0742, a PPARδ agonist, enhances CM proliferation in adult mice, whereas GSK3787, a PPARδ antagonist delivered via aquatic exposure in adult zebrafish, suppresses cardiomyocyte proliferation (Magadum et al. 2017). In contrast, glycolysis inhibition appears detrimental to regeneration. 2-Deoxyglucose (2-DG), a glycolytic inhibitor administered intraperitoneally, consistently suppresses CM proliferation in adult zebrafish across multiple dosing regimens (Honkoop et al. 2019). Atpenin A5, an SDH inhibitor delivered intraperitoneally in neonatal mice, enhances proliferation by reducing mitochondrial ROS production (Bae et al. 2021).

Metabolite analogs provide a targeted means of mimicking endogenous intermediates. Intraperitoneal administration of dimethyl malonate inhibits SDH, promotes CM proliferation in both neonatal and adult mice, whereas dimethyl succinate, a succinate precursor, suppresses proliferation via either intraperitoneal or intravenous routes (Bae et al. 2021). Supplementation with α-KG has similarly been shown to enhance CM proliferation in neonatal and adult mouse hearts (Shi et al. 2024).

Gene delivery platforms enable sustained expression of metabolic regulators, with the delivery route influencing both tissue targeting and efficacy. Adeno-associated virus serotype 9 (AAV9) vectors have been used to deliver *HMGCS2* and *Sphk2* via intramyocardial or tail vein injection, thereby enhancing ketogenesis and nuclear sphingolipid signaling, respectively, and promoting CM proliferation in both neonatal and adult mice (Cheng et al. 2022; Ji et al. 2024). However, the prolonged expression and potential immunogenicity of viral vectors pose translational challenges.

Modified mRNA (modRNA) systems offer a transient and tunable alternative with improved specificity. A CM-targeted modRNA platform incorporating an L7Ae–K-motif translational repression module and miRNA recognition elements (miR-1 and miR-208a) enables selective expression in CMs. Intramyocardial delivery of modRNA encoding PKM2 or glucose-6-phosphate dehydrogenase (G6PD) enhances glycolysis and PPP activity, stimulates mononuclear CM proliferation, and improves cardiac function after myocardial infarction (Magadum et al. 2020). In summary, the route and modality of delivery are critical determinants of therapeutic efficacy in metabolic reprogramming. Continued optimization of spatial targeting, temporal resolution, and combinatorial strategies will be essential for advancing metabolic interventions toward clinical translation.

## Inducing CM proliferation for improving cardiac function in swine models

As mentioned above, significant advancements have been achieved in identifying regenerative growth factors, miRNAs, small molecules, cell-cycle regulators, and transcription factors capable of stimulating CM proliferation. However, translating these discoveries into clinical therapies remains challenging. A crucial bottleneck is the limited availability of robust preclinical data derived from large animal models, which are essential for bridging the translational gap to human clinical applications. Small animal models, including mice and rats, differ considerably from humans in cardiac physiology, disease pathology, and immune responses, thus limiting their predictive accuracy for human therapies.

Consequently, recent efforts have increasingly utilized swine models due to their closer physiological and anatomical similarities to the human heart, making them particularly valuable for investigating regenerative mechanisms involved in cardiac repair. Studies employing swine models have demonstrated therapeutic promise for various regenerative agents in the context of MI and IR injury (Table 4). For example, delivery of Follistatin-like 1 (FSTL1) via an epicardial patch significantly improved cardiac Function and reduced myocardial fibrosis following IR injury in swine models. Adeno-associated virus serotype 6 (AAV6)-mediated delivery of miR-199a enhanced CM proliferation and promoted functional cardiac recovery after MI, though dose-dependent toxicity and safety concerns still need to be addressed (Gabisonia et al. 2019). Similarly, recombinant human Agrin (rhAgrin) demonstrated protective effects against IR injury in porcine hearts (Baehr et al. 2020). Additionally, administration of 4 F preserved myocardial function and limited infarct size in swine MI models (Abouleisa et al. 2022). Notably, utilizing a cardiomyocyte-specific mRNA translation system (SMRTs) to overexpress Cyclin D2 (CCND2) resulted in enhanced CM proliferation and myocardial repair post-infarction (Sun et al. 2023). Moreover, the FDA-approved aminoglycoside antibiotics Paro and Neo, which inhibit the Meis1-Hoxb13 interaction, showed preclinical efficacy in promoting CM proliferation and myocardial regeneration (Ahmed et al. 2024). Collectively, these findings suggest that regenerative factors initially identified in small animal models maintain their pro-regenerative efficacy in swine, providing essential validation for their translational potential.

**Table 4:** Summary of factors inducing CM proliferation and cardiac repair in swine models

Factor	Delivery method	Group	Proliferative index change	Functional index change	Reference
FSTL1	Patch	Sham (*n* = 3); I/R (*n* = 3); IR + Patch (*n* = 1); IR + Patch + FSTL1 (*n* = 1);	/	4 weeks EF: 27% to 40%; Scar area: 410 mm^2^ to 301 mm^2^	Wei et al. 2015a
miRNA-199a	Intramyocardial injection	Empty AAV6; AAV6-hsa-miR-199a-3p	Day 12 Ki67: 2% to 8%; BrdU: 2.2% to 9%; pH3: 0.2% to 0.65%; (*n* = 5)	4 weeks EF: 55% to 65%; (*n* = 1213) Scar size: 28% to 13% (*n* = 8);	Gabisonia et al. 2019
rhAgrin	Antegrade infusion into the LAD	Control (*n* = 8); Single dose (*n* = 6); Dual dose (*n* = 5)	Day 7 Ki67: 10% to 15%; BrdU: 4% to 12%, (*n* = 3)	4 weeks EF: (24.78 ± 1.02)%; (36.81 ± 2.38)%; (41.66 ± 1.79)% Scar size: (1) (21.03 ± 2.16)%; (2) (10.39 ± 1.65)%; (3) (8.51 ± 1.62)%;	Baehr et al. 2020
4F	Intramyocardial injection	LacZ-NIL (*n* = 6); TNNT2-4F-NIL (*n* = 7)	/	4 weeks EF: 25% to 39%; Scar size: 17% to 13%;	Abouleisa et al. 2022
CCND2 modRNA	Intramyocardial injection	Vehicle group (*n* = 5); (nGFP)-CM SMRTs group (*n* = 6); CCND2-CM SMRTs group (*n* = 7)	Day3 Ki67: 1% to 4%; pH3: 0.17% to 0.5% AuroraB: 0.02% to 0.065%; (*n* = 3)	4 weeks EF: 1.41-fold increase; Infarct size: 40% to 20%;	Sun et al. 2023
Paro-Neo	Intravenous (i.v.) injection	Ctr (PBS); Paro-Neo combination	5 weeks Ki67: 0.5% to 3.8%; pH3: 0.25% to 1.0%; AuroraB: 0.02% to 0.28%; (*n* = 3)	5 weeks FS: 38% to 50%; *n* = 7 Scar size: 6% to 3%; *n* = 4	Ahmed et al. 2024

## Conclusions and perspectives

Reactivating CM proliferation in the adult heart remains a central goal of regenerative cardiology. While a growing body of work has identified transcriptional and metabolic regulators that promote dedifferentiation, metabolic rewiring, and cell-cycle reentry, several challenges continue to impede clinical translation. A major hurdle is the lack of clear molecular definition for CMs that transiently reacquire proliferative potential. Although mature CMs can partially dedifferentiate, re-enter the cell cycle, and subsequently redifferentiate, specific markers to track these transient states are still lacking. This limits our ability to determine whether regenerative CMs constitute a distinct progenitor-like population or represent a broader, reversible state within the mature CM pool. Adding to this complexity is the close coupling of structural and metabolic remodeling during CM dedifferentiation. A number of factors, including Hippo-YAP signaling (Heallen et al. 2013; Ikeda et al. 2019; Leach et al. 2017), NRG-ErbB signaling (D'Uva et al. 2015), HMGCS2 (Cheng et al. 2022), the OSKM reprogramming factors (Chen et al. 2021), the 4 F cocktail (Mohamed et al. 2018), and the 5SM compound combination (Du et al. 2022) have been shown to promote CM proliferation by simultaneously driving both phenotypic dedifferentiation and metabolic reprogramming. However, the causal hierarchy between these events remains unclear. Whether metabolic shifts initiate dedifferentiation or occur as a consequence of it is still under investigation. Dissecting this relationship will be essential to identify actionable metabolic nodes that regulate CM fate. Precise spatiotemporal control over regenerative interventions represents another major barrier to clinical translation. Many pro-proliferative signals are broadly expressed and dose-sensitive, and indiscriminate activation can lead to arrhythmias, ectopic proliferation, or pathological remodeling. Some delivery methods still struggle with spatial precision and dosing flexibility. Innovative tools including tissue regeneration enhancer elements (TREEs) and inducible expression systems like Drug-elicitable alternative splicing module (DreAM) offer promising solutions for spatially restricted and temporally reversible gene control (Chen et al. 2025; Yan et al. 2023). Yet, their application in delivering metabolic regulators remains largely unexplored and requires further refinement to meet the demands of cardiac regeneration. Finally, the downstream effectors linking metabolism to transcriptional and epigenetic remodeling remain poorly characterized. Although factors such as HMGCS2 (Cheng et al. 2022), SDH (Bae et al. 2021), and PDK4 (Cardoso et al. 2020) have been shown to promote CM proliferation, their integration into chromatin and gene regulatory networks is not well defined. Future studies integrating metabolic flux profiling, chromatin accessibility assays, single-cell transcriptomics, and temporally resolved functional perturbation will be needed to construct a systems-level view of metabolism-guided cardiac regeneration. Moreover, although several CM proliferation regulators have been tested in porcine models, metabolic profiling in these studies remains scarce. Moreover, most metabolism-based regulators that promote CM proliferation in rodents have yet to be evaluated for efficacy in swine. Given the physiological and metabolic differences across species, validating these targets in large-animal and human-relevant systems is critical for advancing clinical translation. In summary, the field is shifting from discovery to translation. A deeper understanding of how structural dedifferentiation, metabolic rewiring, and cell-cycle activation intersect, and how to modulate them with precision in a clinical setting will be key to realizing the goal of regenerating the injured human heart.

## Data Availability

Not applicable.

## Abbreviations

CM: Cardiomyocyte
miRNAs: MicroRNAs
TCA: Tricarboxylic acid
DPR: Dedifferentiation, proliferation, and redifferentiation
CX43: Connexin 43
MST: Mammalian sterile 20-like kinase
LATS: Large tumor suppressor kinase
SALV: Salvador
TEAD 1: Transcriptional enhanced associate domain 1
OSM: Oncostatin M
Klf1: Krüppel-like factor 1
4F: CDK1, CCNB, CDK4, and CCND
OSKM: Oct4, Sox2, Klf4, and c-Myc
5SM: Phenylephrine hydrochloride, Baricitinib, Harmine, VO-OHpic trihydrate, and AZD3965
YAP: Yes-associated protein
IR: Ischemia-reperfusion
2D2P: Tmsb4x, Tmsb10, Dmd, and Ctnna3
Paro: Paromomycin
Neo: Neomycin
LRRC10: Leucine-rich repeat-containing 10
MBNL1: Muscleblind-like 1
FAO: Fatty acid oxidation
ROS: Reactive oxygen species
P1: Postnatal day 1
PKM2: Pyruvate kinase muscle isozyme 2
PPP: Pentose phosphate pathway
GLUT1: Glucose transporter 1
PDK: Pyruvate dehydrogenase kinase
CPT1B: Carnitine palmitoyltransferase 1B
α-KG: α-Ketoglutarate
KDM5: Lysine (K)-specific demethylase 5
PARP1: Poly (ADP-ribose) polymerase 1
PPAR: Peroxisome proliferator–activated receptor
HMGCS2: 3-Hydroxy-3-methylglutaryl-CoA synthase 2
S1P: Sphingosine-1-phosphate
Sphk2: Sphingosine kinase 2
JMJD3: Jumonji domain-containing protein 3
SDH: Succinate dehydrogenase
PDH: Pyruvate dehydrogenase
DCA: Dichloroacetate
2-DG: 2-Deoxyglucose
AAV9: Adeno-associated virus serotype 9
modRNA: Modified mRNA
G6PD: Glucose-6-phosphate dehydrogenase
FSTL1: Follistatin-like 1
AAV6: Adeno-associated virus serotype 6
rhAgrin: Recombinant human Agrin
SMRTs: Specific mRNA translation system
CCND2: Cyclin D2
TREEs: Tissue regeneration enhancer elements
DreAM: Drug-elicitable alternative splicing module
